# Evaluating the Quality of Health-Related WeChat Public Accounts: Cross-Sectional Study

**DOI:** 10.2196/14826

**Published:** 2020-05-08

**Authors:** Fuzhi Wang, Zhuoxin Wang, Weiwei Sun, Xiumu Yang, Zhiwei Bian, Lining Shen, Wei Pan, Peng Liu, Xingzhi Chen, Lianguo Fu, Fan Zhang, Dan Luo

**Affiliations:** 1 School of Health Management Bengbu Medical College Bengbu China; 2 Innovation Team of Health Information Management and Application Research (BYKC201913) Bengbu Medical College Bengbu China; 3 Project Team of Outstanding Young Teachers Bengbu Medical College Bengbu China; 4 School of Nursing Bengbu Medical College Bengbu China; 5 The General Practice Medical Education and Development Center of Anhui Province Bengbu Medical College Bengbu China; 6 School of Medicine and Health Management Tongji Medical College Huazhong University of Science & Technology Wuhan China; 7 School of Public Health Bengbu Medical College Bengbu China

**Keywords:** health-related WeChat Public Account, HONcode, suitability assessment of material, evaluation, social media, mHealth, app, health information, internet

## Abstract

**Background:**

As representatives of health information communication platforms accessed through mobile phones and mobile terminals, health-related WeChat public accounts (HWPAs) have a large consumer base in the Chinese-speaking world. However, there is still a lack of general understanding of the status quo of HWPAs and the quality of the articles they release.

**Objective:**

The aims of this study were to assess the conformity of HWPAs to the Health on the Net Foundation Code of Conduct (HONcode) and to evaluate the suitability of articles disseminated by HWPAs.

**Methods:**

The survey was conducted from April 23 to May 5, 2019. Based on the monthly (March 1-31, 2019) WeChat Index provided by Qingbo Big Data, the top 100 HWPAs were examined to evaluate their HONcode compliance. The first four articles published by each HWPA on the survey dates were selected as samples to evaluate their suitability. All materials were assessed by three raters. The materials were assessed using the HONcode checklist and the Suitability Assessment of Materials (SAM) score sheet. Data analysis was performed with SPSS version 17.0 (SPSS Inc, Chicago, IL, USA) and Excel version 2013 (Microsoft Inc, Washington DC, USA).

**Results:**

A total of 93 HWPAs and 210 of their released articles were included in this study. For six of the eight principles, the 93 HWPAs nearly consistently did not meet the requirements of the HONcode. The HWPAs certified by Tencent Corporation (66/93, 71%) were generally slightly superior to those without such certification (27/93, 29%) in terms of compliance with HONcode principles. The mean SAM score for the 210 articles was 67.72 (SD 10.930), which indicated “adequate” suitability. There was no significant difference between the SAM scores of the articles published by certified and uncertified HWPAs (*P*=.07), except in the literacy requirements dimension (t_df=97_=–2.418, *P*=.02).

**Conclusions:**

The HWPAs had low HONcode conformity. Although the suitability of health information released by HWPAs was at a moderate level, there were still problems identified, such as difficulty in tracing information sources, excessive implicit advertisements, and irregular usage of charts. In addition, the low approval requirements of HWPAs were not conducive to improvement of their service quality.

## Introduction

With progress of information technology, internet-based new media application platforms are becoming an important resource for the public to obtain health information [1,2]. Compared with traditional health information sources, internet-based online health information (OHI) is widely distributed, abundant, rapidly growing, and diverse in form. A common view about the quality of OHI is that a substantial amount of health information should be produced in a specific medical context, which is often ignored in the internet age, leading to members of the public taking OHI out of context. In addition, many websites often provide links to network information with irrelevant or even fake content for commercial benefit [3]. Therefore, evaluation of the quality of OHI has attracted worldwide attention.

A study conducted at China Renmin University investigated the quality of Chinese health information services with a self-constructed evaluation system and found that privacy protection, information integrity, accessibility, and the platform response of information services were important factors influencing the improvement of user participation [4]. Another study performed at Wuhan University constructed an OHI quality evaluation standard system consisting of two primary indicators, seven secondary indicators, and seven tertiary indicators. In addition, suggestions were proposed for the construction of health information websites and the improvement of the quality of network health information from the perspective of users [5]. These articles used self-compiled evaluation tools to study the quality of OHI, whereas international evaluations of the quality of OHI have mostly used more mature evaluation tools. A review of 70 studies on the quality of OHI in 18 countries showed that the most commonly used evaluation tools globally include DISCERN [6], Health on the Net Foundation Code of Conduct (HONcode) [7], Journal of American Medical Association benchmark [8], and LIDA instrument [9]. Approximately 50% of the 70 studies resulted in completely negative evaluations of OHI, and 27.1% of the studies had both positive and negative evaluations. The main reasons for a negative evaluation included the website organizer and sponsor information were not transparent, the disease description and drug information were not accurate, and the information source and authors’ identities were not disclosed [10]. Another comparative analysis of OHI services in China and the United States showed that the certification of OHI services in China mainly involves official website certification and internet drug information service certification, whereas the United States mainly focuses on HONcode (health information quality certification) and TRUSTe (website safety certification). Although the content of the different website certification systems is similar, the Chinese health website certification system is more focused on evaluating external features such as website structure and services but lacks evaluation of OHI quality [11].

Compared with traditional health information dissemination through a website, WeChat-based health information dissemination is more convenient. Users can “send out” health information to a broad community through WeChat groups through a simple sharing operation via mobile terminals. Therefore, as a new medium of internet information dissemination, WeChat has a unique impact on the dissemination of Chinese OHI.

As online information communication platforms launched by Tencent, WeChat public accounts (WPAs) have been popular in Chinese-speaking communities worldwide. According to the WeChat Data Report 2018 released by Tencent in 2018, the monthly number of active WPAs exceeded 3.5 million, and the monthly number of active fans reached 797 million [12]. WeChat has gained rapid popularity in mainland China, Taiwan, Hong Kong, Macao, and other regions in the world where people of Chinese ethnicity reside, so that WPAs are now an important means of disseminating information [13]. Unlike apps on mobile platforms, WPAs do not differ based on the operating system; both Android and iOS support access to WPAs. In addition, WPAs feature timely information push notifications, content relevant to everyday life, along with light and humorous writing [14].

Health-related WeChat public accounts (HWPAs) are in a stage of rapid development [15]. By April 22, 2019, the top 100 HWPAs according to the WeChat Communication Index (WCI) had published more than 11,000 articles in total, with a total article access count of over 247 million (see Multimedia Appendix 1, downloaded 9:22 am April 22, 2019). HWPAs have an important impact on public health education and health promotion. However, the number of HWPAs is large. From a content perspective, there are official WPAs from medical institutions as well as information service public accounts dedicated to health care, disease rehabilitation, and other health knowledge dissemination. WPA owners include companies, government agencies, nonprofit organizations, and individuals. Although it is convenient for the public to obtain health-related information from WPAs, there is increasing doubt about the quality of the health information released through WPAs [16]. To ensure the authenticity and security of WPAs, the Tencent Corporation provides an authentication service for WPAs. For verified WPAs, the authentication information and WeChat authentication unique identity are displayed in the authentication details of the account. However, certification is not mandatory. Individuals and organizations can still apply for WPAs and release health-related information even without official Tencent certification.

In the past 5 years, a large number of studies have examined the use of WPAs in the fields of health education [17] and health intervention [18,19]; however, there is still a lack of general understanding about the quality of HWPAs and the articles released on such platforms. Accordingly, the aim of this study was to evaluate the HONcode conformity of HWPAs and analyze the suitability of articles posted by HWPAs to provide support for improving the service quality of HWPAs and optimizing the OHI communication environment.

## Methods

### Sample Selection

Many organizations and companies have proposed their own evaluation standards to evaluate the influence of WPAs. One of the most widely used standards is the WCI proposed by Qingbo Bigdata Technology Co Ltd (Beijing, China). Qingbo Big Data is well-known among researchers and policymakers of the new media influence evaluation criterion in China by providing big data technology services for the media, public opinion and industry, and their customers, including the Chinese government, top Chinese news media (eg, Xinhua News Agency, People’s Daily, China National Radio), and large multinational enterprises [20]. The WCI consists of four primary indicators (spread rate of the whole article, average spread rate of each article, title spread rate, and peak spread rate), eight secondary indicators, and a set of calculation formulas for standardized scores [21]. A higher WCI value represents a larger WPA influence. The latest version of the WCI is version 13.0, updated in January 2017.

**Figure 1:**
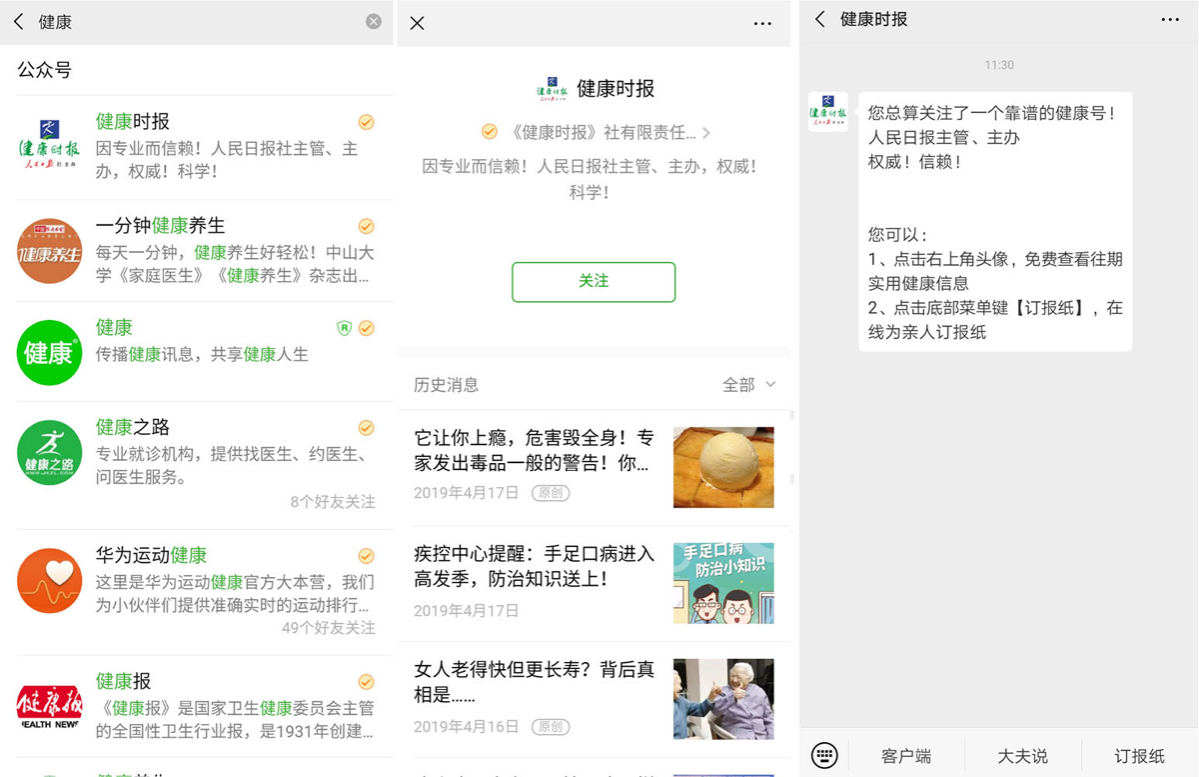
Examples of WeChat public accounts (WPAs). Left: Search results for health-related WPAs (HWPAs) from the keyword 健康 (Health) in the WeChat app. Middle: Client home page of a HWPA. Clicking 关注 (Follow) allows the user to follow the articles published by the HWPA, and clicking the icon and text below allows the user to read the articles published by the HWPA. Right: The HWPA menu bar. The icon below provides information classification support and interaction support for users. Retrieval date: April 22, 2019.

We searched the health category of the WPA monthly list (March 1-31, 2019) provided by Qingbo Big Data. The first 100 HWPAs in the WCI were selected as the survey sample. The exclusion criteria for HWPAs were as follows: (1) commodity sales as the main purpose, (2) religious background, (3) organization service guide, and (4) obvious lack of relationship with health. Finally, 93 HWPAs were included in this study. Some examples of the HWPAs are provided in Figure 1.

**Figure 2:**
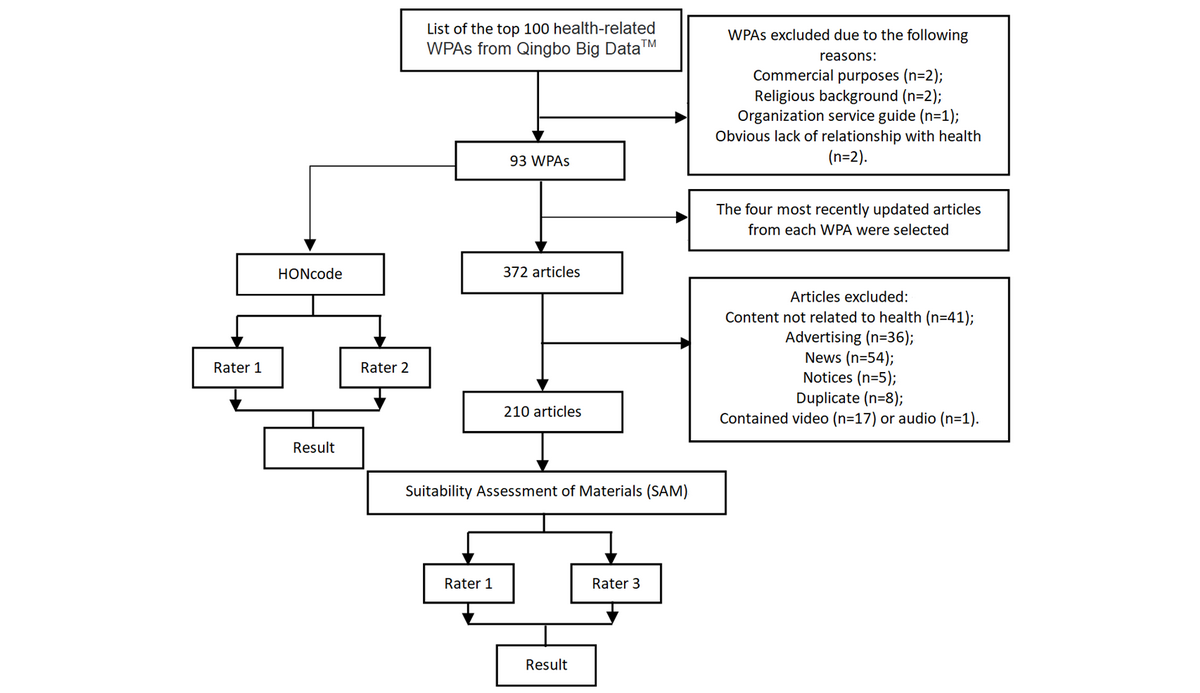
Search and screening flow for health-related WeChat public accounts (HWPAs) and articles. HONcode: Health on the Net Foundation Code of Conduct.

The article sample pool was formed by taking four articles that were newly released by each HWPA on the survey dates (April 23 to May 5, 2019) for a total of 372 articles. The exclusion criteria for articles were as follows: (1) duplicate articles, (2) content not related to health knowledge, (3) advertising/news/notices, and (4) video or audio materials. Finally, 210 articles met the inclusion criteria. Figure 2 illustrates the search and screening flow for the HWPAs and articles.

### Evaluation Tools

To the best of our knowledge, there is no quality assessment tool for WPAs, which are online information dissemination platforms that are similar to websites in terms of information release, content services, and operation modes. Therefore, in this study, the HONcode scale was used as the tool to evaluate HWPA quality specifications. Health on the Net (HON) is an international nongovernmental and nonprofit organization established in Switzerland in 1996. The HONcode for medical and health websites addresses one of the internet’s main health care issues: the reliability and credibility of information. HONcode provides a set of basic ethical standards for website developers to adhere to with respect to the presentation of information, and aims to ensure that readers always know the source and purpose of the data they are reading [7]; it is currently the most widely used code of ethics for the quality of OHI in the world [22]. As online information platforms used to disseminate health knowledge to the public, HWPAs should also follow the HONcode in the construction of their platforms and the determination of their behaviors in OHI dissemination. Therefore, we believe that it is necessary and appropriate to evaluate HONcode compliance for HWPAs.

Although there are many tools available for assessing the quality of OHI, such as the mHONcode, Michigan website evaluation checklist, LIDA scales, DISCERN instrument, and Simple Measure of Gobbledygook (SMOG) readability formula, none of these tools is well suited for assessing the quality of health information on mobile information platforms. The mHONcode is suitable for evaluating health-related apps that run independently on Android or iOS [23], while WPAs are a platform established on the WeChat app. The Michigan checklist and LIDA scales are more suitable for the assessment of browser-based OHI (eg, the content layout of browser pages, use of browser navigation) [24,25], DISCERN was designed to evaluate the quality of online therapeutic information [6], and SMOG lacks support for Chinese-speaking users [26]. Therefore, in this study, we used the Suitability Assessment of Materials (SAM) scale to evaluate the health information released by HWPAs. The SAM scale comprises six dimensions, including content, literacy demand, graphics, layout and typography, learning stimulation/motivation, and cultural appropriateness, which have good reliability and validity and are widely used in evaluating the quality of OHI materials [27]. The SAM scale includes 22 factors for a total of 44 points (100%), with a higher score indicating better suitability. The results are rated on three levels: superior (70%-100%), adequate (40%-69%), and not suitable (0-39%).

### Rating Process

The evaluation was performed by three researchers. Rater 1 (WS) holds a master’s degree in medical informatics and has 7 years of experience in medical information analysis and research. Rater 2 (FW) holds a master’s degree in computer science and a doctorate degree in social medicine, with 8 years of experience in software development. Rater 3 (ZB) holds a doctorate degree and a clinician qualification, with 10 years of clinical experience. Pilot assessments were conducted using 6 HWPAs (3 certified and 3 uncertified). Before the pilot assessment, the three raters carefully studied the simplified Chinese description of the HONcode scale on the HON website and the English version of the SAM sheet to better understand the purpose and significance of each item. The assessment was performed in two steps. First, rater 1 and rater 2 evaluated the HWPAs’ compliance with the principles of the HONcode. Second, rater 1 and rater 3 evaluated the suitability of the articles released by the HWPAs with the SAM scale. To ensure consistency of the evaluation results, the evaluation was carried out with the subdimensions of the scale (ie, the Nth subdimension of all samples was completed, and then the Nth+1 dimension was evaluated). The assessment process was conducted in parallel; that is, two raters independently evaluated the same sample at the same time. For any controversial assessment results, the final results were determined through real-time negotiation.

### Statistical Analysis

Statistical analysis was conducted using the Statistical Package for Social Sciences version 17.0 (SPSS Inc, Chicago, IL, USA) and Excel version 2013 (Microsoft Inc, Washington DC, USA). All values are expressed as the mean (SD). Within-group comparisons of the SAM scores were performed using paired *t* tests. The critical value of significance was determined to be *P*=.05.

## Results

### Characteristics of the Health-Related WeChat Public Accounts

The characteristics of the HWPAs are shown in Table 1. Of the 93 HWPAs, 66 (71%) were officially verified by Tencent Corporation, while 27 (29%) were not. The WCI values ranged between 1479.35 and 877.62 and were divided into three sections: A (the first 20%), 1108.80-1479.35; B (middle 60%), 909.10-1100.52; and C (the last 20%), 877.62-907.74. The owners of certified HWPAs were mainly companies, whereas uncertified HWPAs were mainly owned by individuals. With respect to the type of information released, the certified and uncertified HWPAs were similarly dominated by comprehensive information, followed by traditional Chinese medicine (TCM).

**Table 1:** Summary of the descriptive and frequency statistics for the final sample of WeChat public accounts.

Characteristic		Certified (N=66)		Uncertified (N=27)
**Subject classification, n (%)**				
	Company	53 (80)	3 (11)	
	NGO^a^	12 (18)	1 (4)	
	Individual	1 (2)	23 (85)	
**WCI^b^**				
	Section A	17 (26)	3 (11)	
	Section B	35 (53)	19 (70)	
	Section C	14 (21)	5 (19)	
**Type of content**				
	Traditional Chinese medicine	5 (7)	7 (26)	
	Rational diet	2 (3)	3 (11)	
	Sports and health	2 (3)	3 (11)	
	Mental health	1 (2)	0 (0)	
	Medical scientific research	2 (3)	0 (0)	
	Health of key population	5 (8)	0 (0)	
	Comprehensive information	49 (74)	14 (52)	

^a^NGO: nongovernmental organization.

^b^WCI: WeChat Communication Index.

### Health on the Net Foundation Code of Conduct Conformity

The HONcode compliance of the 93 HWPAs is shown in Multimedia Appendix 2. Although certified HWPAs were slightly more compliant than uncertified HWPAs, the compliance of HWPAs with the HONcode principles was generally not ideal, especially regarding the six principles of privacy, attribution, justifiability, transparency, financial disclosure, and advertising policy. For the remaining two principles, the compliance was also uneven. Although most HWPAs provided information on the content providers, they seldom verified the qualifications of these providers. In addition, most of the HWPAs did not state that the health-related information they provided was intended to support rather than replace medical decisions, and they did not clearly identify the user groups they were targeting.

### Suitability of Articles From WeChat Public Accounts

Among the six subdimensions of the SAM scale, layout and typography scored the highest (4.83/6 points), and graphics (5.167/10 points) and learning stimulation (3.68/6 points) scored the lowest. The remaining three subdimensions scored somewhere in between (7.22/10 points and 3.02/4 points), closer to the typical SAM score (34/44 points, 77%). There were 89 (42.4%) articles (mean 77.58, SD 5.496) that met the criteria for “superior” suitability as established by the SAM scale, 118 (56.2%) articles (mean 61.06, SD 7.014) that met the criteria for “adequate” suitability, and 3 (1.4%) articles (mean 38.64, SD 2.143) that were evaluated as “not suitable”. The mean SAM score was 67.70 (SD 10.93), indicating “adequate” suitability. The descriptive statistics of the SAM scale are shown in Table 2.

The results of the *t* test (Table 3) indicated that there were no significant differences in the SAM scores of the articles released by HWPAs with and without Tencent certification, except for the literacy requirements dimension.

**Table 2:** Descriptive statistics of the Suitability Assessment of Materials scale (N=210).

Item		Not suitable		Adequate		Superior
**Content, n (%)**						
	Purpose		0 (0)		23 (10.9)	187 (89.1)
	Content topics		1 (0.5)		96 (45.7)	113 (53.8)
	Scope		3 (1.4)		117 (55.7)	90 (42.9)
	Summary/review		51 (24.3)		100 (47.6)	59 (28.1)
**Literacy demand, n (%)**						
	Reading grade level		60 (28.6)		105 (50.0)	45 (21.4)
	Writing style		28 (13.3)		67 (31.9)	115 (54.8)
	Vocabulary		29 (13.8)		109 (51.9)	72 (34.3)
	Context		1 (0.5)		26 (12.4)	183 (87.1)
	Advanced organizers		13 (6.2)		14 (6.7)	183 (87.1)
**Graphics, n (%)**						
	Cover graphic		42 (20.0)		107 (51.0)	61 (29.0)
	Type of illustrations		24 (11.4)		95 (45.3)	91 (43.3)
	Relevance of illustrations		41 (19.5)		94 (44.8)	75 (35.7)
	List, tables, graphs, charts		76 (36.2)		73 (34.8)	61 (29.0)
	Captions		103 (49.1)		74 (35.2)	33 (15.7)
**Layout and typography, n (%)**						
	Layout		0 (0)		96 (45.7)	114 (54.3)
	Typography		5 (2.4)		89 (42.4)	116 (55.2)
	Subheadings		20 (9.5)		11 (5.2)	179 (85.2)
**Learning stimulation/motivation, n (%)**						
	Interaction		139 (66.2)		66 (31.4)	5 (2.4)
	Modeling of behaviors		6 (2.9)		48 (22.9)	156 (74.3)
	Motivation		18 (8.6)		47 (22.4)	145 (69.1)
**Cultural appropriateness, n (%)**						
	Cultural match		1 (0.5)		17 (8.1)	192（91.4）
	Cultural image and examples		34 (16.2)		119 (56.7)	57 (27.1)

**Table 3:** Evaluation scores of articles on the WeChat public accounts (mean, SD).

SAM^a^ item	Certified	Uncertified	*t*	*P* value
Content	5.94 (1.244)	5.70 (1.049)	–1.362	.18
Literacy demand	7.06 (1.764)	7.70 (1.612)	–2.418	.02
Graphics	4.98 (2.683)	5.72 (2.582)	1.777	.08
Layout and typography	4.77 (1.181)	5.00 (1.019)	1.263	.21
Learning stimulation, motivation	3.70 (1.135)	3.62 (1.023)	–0.466	.64
Cultural appropriateness	2.99 (0.734)	3.11 (0.670)	1.155	.25
Total Score	29.44(4.993)	30.85(4.190)	1.847	.07

^a^SAM: Suitability Assessment of Materials.

## Discussion

### Principal Findings

The HWPAs had overall low HONcode conformity. Although the suitability of health information released by HWPAs was at a moderate level, there were still problems identified such as difficulty in tracing information sources, excessive implicit advertisements, and irregular usage of charts.

HONcode certification was significantly correlated with website quality as measured by DISCERN [28]. However, this survey found that the HONcode compliance of HWPAs was low, which is similar to the HONcode compliance of nonChinese health websites such as those on urinary diseases [29] and Ebola [30]. By analyzing the certificate information of HWPAs, we found that most of the certified HWPA owners were companies. These companies also have their own websites that are accessed through internet browsers in addition to the HWPAs, and the articles they pushed through HWPAs were also published on their websites. After further checking these Web page-based websites, we found that none of the websites included in this survey had HONcode certification. Although relevant studies on the HONcode compliance of nonChinese health websites have reported some cases of missing HONcode certification [28,31], the results of this survey are clearly more negative. The main approach to monitor the quality of online health information in China is to check the business scope and content of the network information platform according to relevant laws and regulations. This approach emphasizes the binding force of the legitimacy of the network information platform and the external monitoring of the online information platform. HONcode is not a strict regulation but is rather an ethical code for the release of online information that emphasizes the constraints on online information providers from the perspective of professional spirit, moral conscience, and internal monitoring of the network information platform [32]. In this survey, we found that the business entities of HWPAs paid more attention to compliance with laws and regulations but paid insufficient attention to the ethical standards for online information, such as privacy, attribution, justification, or transparency. This may be the main reason for the low HONcode conformity of HWPAs.

We also found a lack of appropriate mechanisms for monitoring the backgrounds of the HWPAs. Regardless of whether the HWPAs were operated by individuals or by organizations, there was no requirement to provide any medical qualification-related certification materials to apply for a WPA [33]. Although some organizations (such as hospitals, government agencies, and medical-related media) have business licenses, we can only infer whether they have medical-related qualifications [34] or internet content provider qualifications [35]; however, some trading companies, advertising companies, associations, and other organizations have also received Tencent’s official WPA certification (see Multimedia Appendix 3). For personal applicants, as long as they submit identification information (eg, an identity card), a cell phone number, and a bank card tied to a WeChat ID, they can apply to establish their own HWPAs without the need to provide any proof of medical qualifications [33].

In addition, it is common to search for WPAs by name. However, the names of many WPAs were not matched to their WeChat IDs. For example, we searched for a WPA named 中医养生 (Traditional Chinese Medicine and Healthcare), and 11 results were returned. Although each WPA has a unique WeChat ID, it is difficult for consumers to remember the IDs or to distinguish among the WPAs using their IDs.

Regarding the overall evaluation, the HWPA health information suitability was determined to be at the “moderate” level, which is similar to the published OHI suitability evaluation results [36,37]. However, regarding literacy demand and cultural appropriateness, the HWPA scores were significantly better than those of some nonChinese OHI evaluation results [28]. This may be because, compared with China with a relatively stable cultural environment, countries in Europe and America have more immigrants and face more problems such as cultural assimilation [38], income disparity [39], and disease burden [40]. The resulting cultural and linguistic differences inevitably lead to differences in people’s health-related behavior [41] and understanding of OHI [42]. On the one hand, this requires website owners to consider more acculturation factors when publishing health information. On the other hand, it creates higher requirements for users’ cultural literacy [43].

In terms of scoring dimensions, most of the health-related articles published by HWPAs had friendly cover pictures and attractive titles that clearly described the purpose of the article, had a good layout and typography, and were culturally suitable. However, the use of charts was not standardized, and the lack of charts used as illustrations was a common problem. More than half of the articles included pictures with little relevance to the content of the articles or even negative exaggerations and stereotypical cultural characteristics. In addition, in terms of the vocabulary used, readers would have little difficulty reading the articles, but articles related to TCM generally used professional terms. Although knowledge of TCM, as part of the national traditional culture, can be expected of most Chinese residents, there was still a large amount of content that would be difficult for readers to understand [44], indicating a higher level of literacy required to understand the articles.

In addition, compared with the traditional mode of health information dissemination through Web pages, WPAs require simple operations to share information, and the integrated payment functions in WeChat, Alipay, and other apps only require a few steps to complete commodity purchases and even provide short-term interest-free loan and installment repayment services. Therefore, article copying and implicit advertisements were common problems of the HWPAs. We randomly selected several articles, which were searched using the Baidu search engine, and found that some articles were posted on different websites; the same results were obtained for several articles marked as “original” on the HWPAs. Although most websites cited their sources, the sources indicated for the same articles on different websites were often inconsistent. This might explain why there was no significant difference in the results of the evaluation of the suitability of articles released by certified and uncertified HWPAs. Advertisements were usually embedded in the text in the form of pictures or articles introducing sales information for products in the form of an article summary. We assessed the accuracy of the health knowledge disseminated through several articles containing implicit advertisements and found no medicine-related errors. However, such implicit advertisements might still cause undesirable subjective feelings regarding user access, reduce consumers’ trust in the content of the articles, or mislead consumers regarding healthy behaviors [45].

### Implications

New media has become an important resource for the public to seek health information. As representatives of Chinese health information communication platforms accessed through mobile phones and mobile terminals, HWPAs have a large consumer base in the Chinese-speaking world [46]. We suggest that the owners of HWPAs should follow the HONcode to guide and improve the construction of their HWPAs and strengthen the quality control of the OHI they publish. At the same time, as the manager of WeChat public account platforms, the Tencent Corporation should strengthen the qualification requirements for applying for an HWPA and strengthen the supervision of the health-related content released by WPAs to avoid the occurrence of another “Wei Zexi incident” [47] on WPA platforms. In addition, we suggest that related research institutions formulate targeted norms for the construction of mobile OHI platforms.

### Limitations

Several limitations of this study are apparent. First, there are many evaluation indices of WPAs, but there is a lack of horizontal comparison of these indices. In this study, we chose the WCI proposed by Qingbo Bigdata as the ranking basis for the influence of WPAs, which may have resulted in selection bias. Second, as a set of principles that health websites should follow, the HONcode is important in guiding the construction of HWPAs. However, a few indicators were not well targeted to mobile platforms, which may have reduced the validity of the assessment. Third, the Chinese nation is a multiethnic group, and some ethnic minorities have their own spoken and written languages. However, due to the WCI ranking system, the content on the HWPAs in this survey was all in simplified Chinese. Finally, all evaluations were influenced by the researchers who conducted them, and the results of their evaluations may differ from consumers’ feelings. In view of the above limitations, the conclusions of this study are preliminary and should be carefully interpreted.

### Conclusions

We found that HWPAs had low compliance with the HONcode. Although the suitability of the articles released by HWPAs was at a moderate level, there were still problems identified, such as difficulty in tracing the sources of information, excessive implicit advertisements, and the irregular usage of charts. Moreover, low approval requirements for applications to obtain an HWPA are not conducive to improving the service quality of HWPAs.

## Appendix

Multimedia Appendix 1 WCI ranking data for WPAs (March 1-31, 2019).

Multimedia Appendix 2 Descriptive statistics of HONcode conformity (n, %).

Multimedia Appendix 3 Raw data of HWPAs rating scale.

## Abbreviations

HONcode: Health on the Net Foundation Code of Conduct
HWPA: health-related WeChat public account
OHI: online health information
SAM: Suitability Assessment of Material
SMOG: Simple Measure of Gobbledygook
TCM: traditional Chinese medicine
WCI: WeChat Communication Index
WPA: WeChat public account
