# Supplementary material for: Evaluating the Quality of Health-Related WeChat Public Accounts: Cross-Sectional Study
Source: JMIR Mhealth Uhealth. 2020 May 8;8(5):e14826. doi: 10.2196/14826 (PMC7244997; doi:10.2196/14826)
Supplement: Multimedia Appendix 2 [file mhealth_v8i5e14826_app2.docx]

| **Principle** | **Item** | **Option** | **Results** | |
| --- | --- | --- | --- | --- |
|  |  |  | **Certified**  **(n=66)** | **Uncertified**  **(n=27)** |
| **Principle 1:**  Authority | 1. Gives general information about the organization or person responsible for the site’s actions and content, as well as information about the editor or lead author. | Yes, the name of the editor or author is given. | 61 (92) | 18 (67) |
|  |  | No. | 5 (8) | 9 (33) |
|  | 2. Accredits medical health information or advice providers. | Provided by professionals. | 13 (20) | 1 (4) |
|  |  | Provided disclaimers for information provided by non-medical professionals. | 2 (3) | 2 (7) |
|  |  | Some medical health information lacking citations. | 51 (77) | 24 (89) |
| **Principle 2:**  Complementarity | 3. States that the health information released is intended to support rather than replace the doctor-patient relationship. | Yes. | 20 (30) | 3 (11) |
|  |  | No. | 46 (70) | 24 (89) |
|  | 4. States the purpose or motive for creating the site. | Yes. | 63 (95) | 26 (96) |
|  |  | No. | 3 (5) | 1 (4) |
|  | 5. States the target user group. | Yes. | 24 (36) | 4 (15) |
|  |  | No. | 42 (64) | 23 (85) |
| **Principle 3:**  Privacy | 6. States the privacy/confidentiality policy of the health information. | Yes. | 13 (20) | 5 (19) |
|  |  | No. | 53 (80) | 22 (81) |
|  | 7. States that the site complies with the privacy laws of the country where it is located. | Yes. | 9 (13) | 1 (4) |
|  |  | No. | 1 (2) | 0 (0) |
|  |  | Unknown. | 56 (85) | 26 (96) |
| **Principle 4:**  Attribution | 8. Gives the final modification date. | Yes, the whole site. | 2 (3) | 0 (0) |
|  |  | Yes, all web pages that contain medical health information. | 17 (25) | 8 (30) |
|  |  | Yes, all web pages. | 3 (5) | 1 (4) |
|  |  | No. | 44 (67) | 18 (66) |
|  | 9. Notes the sources or external information resources. | Yes, but the external resources are not specified. | 4 (6) | 2 (7) |
|  |  | Yes, hyperlinks to external resources are given. | 19 (29) | 8 (30) |
|  |  | Yes, a reference directory for external resources is given. | 58 (88) | 20 (74) |
|  |  | No, the website content is all original, written by the website editing team. | 1 (2) | 3 (11) |
| **Principle 5:**  Justifiability | 10. Provides a summary of the treatment regimen, products or the efficacy of the service. | Yes, all of the information is well-founded, and the references for scientific research findings and/or published literature are clearly identified. | 12 (18) | 2 (8) |
|  |  | Yes, the information is based on the owner’s own research or opinion. | 4 (6) | 9 (33) |
|  |  | No. | 50 (76) | 16 (59) |
| **Principle 6:**  Transparency | 11. Provides an email address or link for contact. | Yes. | 15 (23) | 4 (15) |
|  |  | No. | 51 (77) | 23 (85) |
| **Principle 7:**  Financial disclosure | 12. Declares the source of working capital. | Yes. | 2 (3) | 0 (0) |
|  |  | No. | 64 (97) | 27 (100) |
| **Principle 8:**  Advertising policy | 13. Has clearly labelled advertisements. | One section describes the advertising strategy. | 2 (3) | 0 (0) |
|  |  | Editorial content and advertising is clearly separated. | 6 (9) | 5 (19) |
|  |  | There is no explanation for the banner. | 58 (88) | 20 (74) |
|  |  | All advertising slogans are clearly marked with the word "advertisement". | 11 (17) | 7 (26) |
|  |  | The advertisement is not labelled in that way. | 41 (62) | 16 (59) |
|  | 14. Declares link exchanges. | Yes, there is a statement that accurately describes the relationship between the site and other sites. | 0 (0) | 0 (0) |
|  |  | Yes, there is a statement that clarifies the relationship between the site and other sites by mentioning the business benefits gained from these exchanges. | 0 (0) | 0 (0) |
|  |  | Yes, but there is no specific description of the policy. | 22 (33) | 7 (26) |
|  |  | No. | 44 (67) | 20 (74) |
|  | 15. Does not display advertising. | Expressly states that the site does not accept or display advertising. | 0 (0) | 0 (0) |
|  |  | There is no such statement. | 66 (100) | 27 (100) |
